# DNetDB: The human disease network database based on dysfunctional regulation mechanism

**DOI:** 10.1186/s12918-016-0280-5

**Published:** 2016-05-21

**Authors:** Jing Yang, Su-Juan Wu, Shao-You Yang, Jia-Wei Peng, Shi-Nuo Wang, Fu-Yan Wang, Yu-Xing Song, Ting Qi, Yi-Xue Li, Yuan-Yuan Li

**Affiliations:** Shanghai Center for Bioinformation Technology, Shanghai, 200235 P.R. China; Key Laboratory of Systems Biology, Institute of Biochemistry and Cell Biology, Shanghai Institutes for Biological Sciences, Chinese Academy of Sciences, Shanghai, 200031 P.R. China; Shanghai Industrial Technology Institute, 1278 Keyuan Road, Shanghai, 201203 P.R. China; Shanghai Engineering Research Center of Pharmaceutical Translation, 1278 Keyuan Road, Shanghai, 201203 P.R. China

**Keywords:** Human disease network, Disease similarity, Dysfunctional regulation mechanism, Differential coexpression analysis, Differential regulation

## Abstract

Disease similarity study provides new insights into disease taxonomy, pathogenesis, which plays a guiding role in diagnosis and treatment. The early studies were limited to estimate disease similarities based on clinical manifestations, disease-related genes, medical vocabulary concepts or registry data, which were inevitably biased to well-studied diseases and offered small chance of discovering novel findings in disease relationships. In other words, genome-scale expression data give us another angle to address this problem since simultaneous measurement of the expression of thousands of genes allows for the exploration of gene transcriptional regulation, which is believed to be crucial to biological functions. Although differential expression analysis based methods have the potential to explore new disease relationships, it is difficult to unravel the upstream dysregulation mechanisms of diseases. We therefore estimated disease similarities based on gene expression data by using differential coexpression analysis, a recently emerging method, which has been proved to be more potential to capture dysfunctional regulation mechanisms than differential expression analysis. A total of 1,326 disease relationships among 108 diseases were identified, and the relevant information constituted the human disease network database (DNetDB). Benefiting from the use of differential coexpression analysis, the potential common dysfunctional regulation mechanisms shared by disease pairs (i.e. disease relationships) were extracted and presented. Statistical indicators, common disease-related genes and drugs shared by disease pairs were also included in DNetDB. In total, 1,326 disease relationships among 108 diseases, 5,598 pathways, 7,357 disease-related genes and 342 disease drugs are recorded in DNetDB, among which 3,762 genes and 148 drugs are shared by at least two diseases. DNetDB is the first database focusing on disease similarity from the viewpoint of gene regulation mechanism. It provides an easy-to-use web interface to search and browse the disease relationships and thus helps to systematically investigate etiology and pathogenesis, perform drug repositioning, and design novel therapeutic interventions.

Database URL: http://app.scbit.org/DNetDB/#.

**Electronic supplementary material:**

The online version of this article (doi:10.1186/s12918-016-0280-5) contains supplementary material, which is available to authorized users.

## Background

Disease similarity study has been attracting more and more attention in recent years, because understanding how the diseases are related to each other not only provides new insights into disease taxonomy, etiology, but also helps to perform drug repositioning and drug target identification [1]. Previous studies have been able to explore disease similarity, i.e., disease relationship, from clinical manifestations [2–6], electronic medical records [7–10], disease-related genes [1, 11], miRNA [12], proteins [13] or pathways [14], chemical fragments [15], microbiota [16], disease-related metabolic reactions [17], disease-related differentially expressed genes [18–20] and multi-types of data [9, 19, 21]. Accordingly, there have been several disease relationship databases, such as 1) the Human Phenotype Ontology (HPO) which shows phenotypic similarities of diseases based on shared clinical synopsis features extracted from OMIM [22]; 2) the Comparative Toxicogenomics Database (CTD) which includes a ‘DiseaseComps’ section to show similar disorders via a) chemical or gene connections, and b) marker/mechanism or therapeutic associations [23]; 3) Malacards which presents disease relationships based on similar medical vocabulary concepts and common disease-related genes [24]; 4) DisGeNET which includes gene-disease associations and disease-disease associations,they evaluate disease-disease associations on shared genes [25]. The above databases heavily depend on prior knowledge such as manifestations, disease related genes, and so on, therefore they provide limited chance of discovering novel disease relationships. Fortunately, the rapidly accumulated high-throughput data such as transcriptomic data offer more possibilities of extracting novel disease relationships. There have been some works which estimated disease similarities through differential expression analysis [18, 20]. However, it has been widely accepted that differential expression analysis have little power to find out dysfunctional regulatory relationships underlying pheonotypes; at this point, differential coexpression analysis method have been proved to be more powerful [26].

In our previous study, we developed a series of differential coexpression analysis and differential regulation analysis methods, which could identify differentially coexpressed genes and links (DCGs and DCLs) in a quantitative way, and furthermore extract differentially regulated genes and links (DRGs and DRLs) via integrating transcriptional regulation knowledge [26–29]. In this work, we explored disease similarities in terms of dysfunctional regulation mechanisms by using our differential coexpression analysis method. We first obtained 86 GSE (short for GEO series) datasets corresponding to 108 diseases, and then calculated differential coexpression value (dC value) of every genes for each disease by using DCp algorithm [28, 29]. The dC value at gene level was then converted into dC at pathway level. The disease similarity was finally estimated as the partial Spearmen correlation coefficients of pathways’ dC values of the two diseases. By applying a permutation test, a total of 1,326 significant disease relationships at a *p*-value threshold of 0.05 (FDR = 20.91 %) were identified among 108 diseases, which constructed the basic information of our human disease network database (DNetDB). The detailed procedure was reported in a companion paper [30]. Meanwhile, in order to facilitate scientists in this field to identify disease relationships with our strategy, the computational method was developed into an R package, DSviaDRM [31].

In DNetDB, 1,326 disease relationships among 108 diseases, 5,598 pathways, 7,357 disease-related genes and 342 disease drugs are recorded. The potential common dysfunctional regulation mechanisms (i.e. shared DCGs, DRGs), common disease-related genes and common drugs shared by disease pairs are also included. All the involved GSEs, genes, pathways or drugs can be linked to their original databases, and all data in DNetDB can be downloaded as well.

## Construction

### Gene expression dataset

We obtained 954 GSE datasets using a unique platform, GPL96, from NCBI Gene Expression Omnibus (GEO, http://www.ncbi.nlm.nih.gov/geo/) [32] during the period from 2003 to 2013. By setting the following three rules: 1) more than five samples in health condition and disease condition respectively 2) samples coming from fresh organs (excluding cell lines) 3) samples in health condition and disease condition coming from the same organs or cells, 106 GSEs were remained. Then, we controlled the quality of raw data (CEL files) of each sample and removed low quality samples using affy [33] and affyQCReport [34] packages in R. Subsequently, we maintained 86 GSEs for 89 disease states which involve 3,068 disease samples and 1,335 health samples. In those GSEs, we merged multi-GSEs if they studied on the same disease and the same tissue. And we split single GSE if it contained multi diseases or multi tissues. Finally, the disease number was expanded from 89 to 108 because 11 out of the original 89 diseases involved two or more tissues. The 108 diseases were written as “disease - tissue”, for example, “Type 2 diabetes – liver”. Besides, a total of 24 sample types were involved.

### Pathway data

Molecular signature database (MSigDB), a collection of annotated gene sets, contains 7 major collections [35]. We selected 6,176 pathways from 2 collections of MSigDB v4.0: 1) curated gene sets which collected from public pathway databases (such as BIOCARTA. REACTOME, KEGG, etc.), publications in PubMed and knowledge of domain experts, 2) GO gene sets which contained GO biological process, GO cellular component and GO molecular function. We excluded some pathways whose members were not detected by GPL96 platform significantly using the binomial probability model to reduce the influence of missing data. Consequently, we kept 5,598 pathways covering a total of 21,003 unique genes.

### Disease-related gene and drug

We collected 7,357 genes which known to be associated with 101 out of 108 diseases from Genetic Association Database (GAD) [36], Online Mendelian Inheritance in Man (OMIM) [37], Human Gene Mutation Database (HGMD) [38] and human single amino acid variants (SAV) of UniProt. We also collected 342 disease drugs for 83 diseases, which downloaded from DrugBank [39].

### Identification of disease similarity

**Fig. 1:**
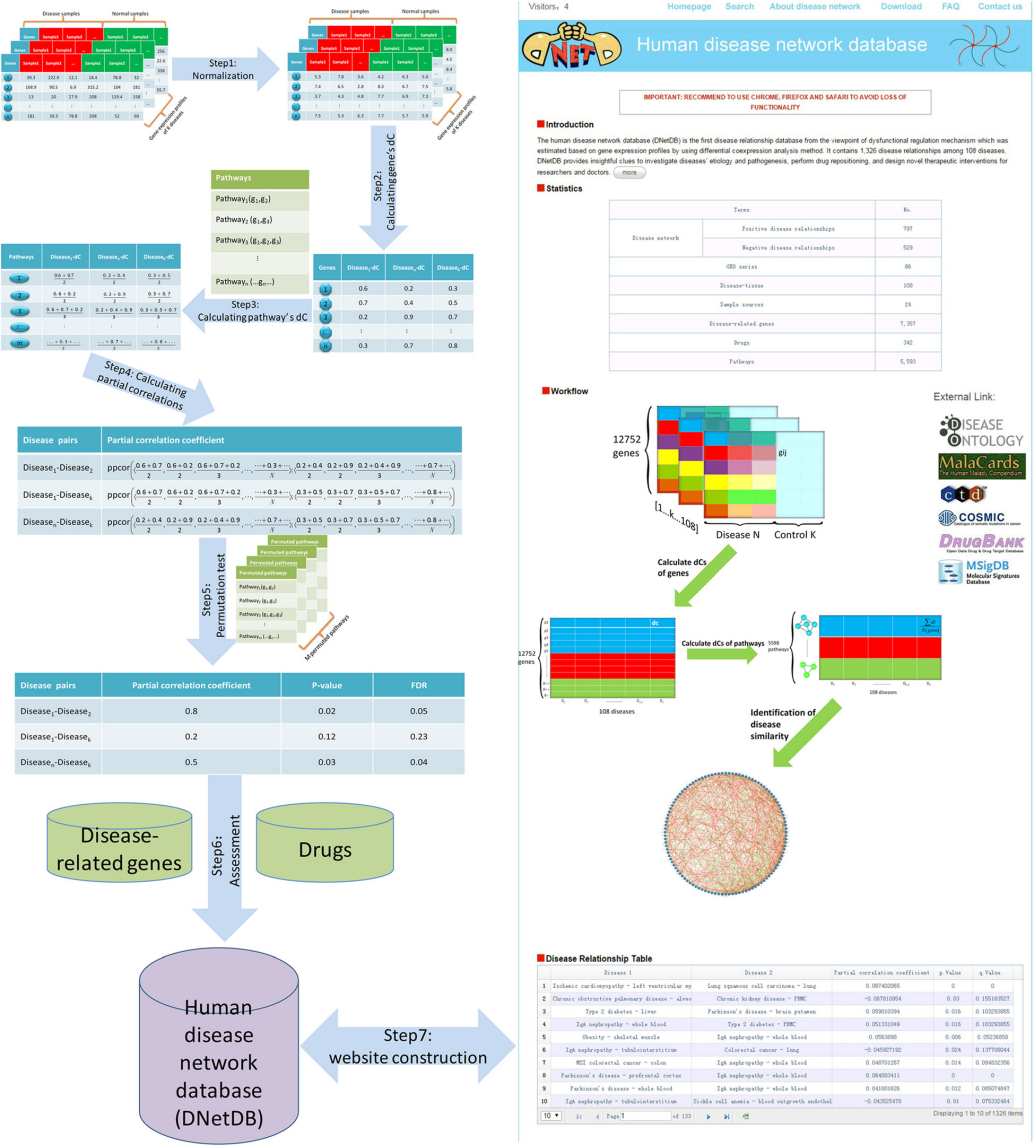
Overview of the DNetDB architecture

Left part of Fig. 1 shows the workflow of disease similarity algorithm. Firstly, for 108 datasets with disease samples and corresponding health samples, we normalized the gene expression values using MAS5.0, respectively. Then differential coexpression value (dC) of each gene for each disease state was obtained by using Differential Coexpression profile (DCp) method of DCGL v2 which quantified the degree of gene correlation change [27–29]. Accordingly, we could obtain a dC matrix in which row denotes gene, and column denotes disease. Then we assigned pathways’ dCs to be the average dC of their component genes, resulting in another dC matrix in which row denotes pathway and column denotes disease. That is, a vector of pathways’ dCs for each disease was obtained (please see the Additional file 1 for the details of differential coexpression analysis). Finally, we used the R package, ppcor, to account the partial Spearman correlation coefficient of pathways’ dCs between any two diseases as the disease similarity.

In order to evaluate the statistical significance of disease correlations, we performed a permutation test, in which we randomly re-assigned the affiliation of gene to pathway with three numbers unchanged: 1) the number of pathways, 2) the number of pathways’ component genes and 3) the number of pathways a given gene belongs to, then accounted the pathways’ dCs and calculated the partial correlations coefficients using permuted data. This procedure was repeated 500 times, and a large number of partial correlation coefficient statistics form an empirical null distribution. The p-value and FDR value for each disease pair can then be estimated. Following above processing, by the cutoff *p* < 0.05 (FDR < 20.91 %), we got 1,326 significant disease relationships among 108 diseases including 797 positive disease relationships and 529 negative relationships. It is noted that when disease A and A’ form a negative link, the patient with disease A tends to be protected from having disease A’ and vice versa, which is probably due to the inversely regulated biological processes involved in the two negatively correlated diseases [20]. We also propose that an anti-A drug may have an undesired property of inducing disease A’ when the drug is inversing its target processes (Additional file 1) [30].

According to the basic understanding that similar diseases tend to share similar pathogenesis, and thus have the potential to be treated by common drugs, we tested whether the 1,326 disease pairs significantly share disease-related genes or drugs with Hypergeometric test [18]. A total of 1,119 out of 1,326 disease pairs in the disease network could be associated with known disease genes; similarly, 745 out of 1,326 disease pairs correlated to known drugs. The Hypergeometric tests for the 1,119 pairs and 745 pairs indicated that 910 of 1,119 disease pairs (81 %) significantly shared known disease genes, and 348 of 745 disease pairs (47 %) significantly shared drugs, both at a *p*-value threshold of 0.05. And the proportion, 81 % and 47 %, are also significantly higher than in random (*p*-values were 0.009 for disease-related genes and 0.023 for disease drugs with one-sided Fisher exact test) [30].

In order to study the consistency of 1,326 disease pairs with previous knowledge on disease classification, we compared the DNetDB with traditional disease classification, including MeSH, ICD-10 or DO databases. If a disease relationship is not located in an common disease category in traditional disease classification, this relationship is marked as “Ture” in the feature of “novel relationship” (Additional file 1).

## Content

Since a total of 1,326 disease relationships are inferred based on the similarity of differential coexpression features of genes, it offers us the possibility to explore the common dysfunctional regulation mechanisms underlying disease pairs by extracting common DCGs and DRGs shared by disease pairs. Therefore, we sorted out common DCGs and DRGs for each disease relationship. The pathways significantly enriched by the shared DCGs are termed as the shared pathways of disease pairs.

In total, our DNetDB database is composed of two parts, basic descriptions of the 108 diseases and information of the 1,326 disease relationships.

### Basic information of diseases

The basic information of the 108 diseases includes disease definition, abbreviation for disease name, six types of disease IDs (including disease IDs in DO, MeSH, ICD10, ICD9, OMIM and NCIt databases), GSE IDs, disease sample tissue and its abbreviation, samples size (the number of disease samples and health samples), disease-related genes and drugs. Overall, DNetDB involves 108 diseases, 5,598 pathways, 7,357 disease-related genes and 342 disease drugs.

### Information of disease relationships

DNetDB records all 1,326 disease relationships with eleven items: 1) partial correlation coefficients, 2) *p*-values of permutation test, 3) FDR values of permutation test, 4) shared DCGs, 5) shared pathways, 6) shared DRGs, 7) 3,762 shared disease-related genes, 8) p-values of the hypergeometric test for shared disease genes, 9) 148 shared drugs, 10) p-values of the hypergeometric test for shared drugs, and 11) comparison with traditional disease classification.

It is noted that we identified 529 negative disease relationships. When two diseases form a negative link, the patient with one disease tends to be protected from the other disease and vice versa. This is probably attributed to the inversely regulated biological pathways involved in the negatively correlated diseases [20]. For example, in both Hu et al.’s work [20] and ours, Muscular dystrophy was negatively correlated to some cancers, which is consistent with the observations that Muscular dystrophy inhibits cell overgrowth while cancer activates it. We furthermore proposed that a drug targeting a certain disease may have an undesired impact of inducing its negatively correlated diseases when the drug is inversing the relevant processes. Taking Crohn’s disease and its drug, infliximab, as an example, Crohn’s disease proved to be negatively connected with T-cell source of chronic lymphocytic leukemia (correlation coefficient −0.15, at top 5 %) and Melanoma (correlation coefficient −0.05, at top 50 %) in our work [30]; infliximab, an antibody against TNF-α, is used for treatment of inflammatory bowel disease (IBD) such as Crohn’s disease [40]. In 2006, FDA issued a warning for infliximab considering its potential association with the development of Hepatosplenic T-cell lymphoma, a subtype of T-cell source of chronic lymphocytic leukemia [41]. More details were provided by our companion research article [30]. We believe that the differential coexpression inforamtion of these negatively correlated diseases helps to explore the mechanisms and improve the therapeutic applications.

## Utility

A user-friendly web interface of DNetDB was developed. All the data were stored and managed in MySQL database. The interface provides six modules: Homepage, About disease network, Search, Download, FAQ and Contact us, which are illustrated as follows.

### Homepage

Homepage module displays basic statistics about DNetDB including disease number, disease relationship number, sample source number and GSE number, the workflow of disease similarity algorithm and the illustration of the whole disease network involving all 1,326 disease relationships. In order to facilitate users to obtain more information, the homepage provides hyperlinks to the collections of source data, such as disease databases (DO, Malacards), gene database (Genecards), and pathway database (KEGG), and drug database (Drugbank) (Fig. 1).

### About disease network, FAQ and Contact us

The motivation, data collection, data processing, evaluation method and application scope of DNetDB are presented in About disease network module. The FAQ module addresses those frequently asked questions about the database. If FAQ does not answer users’ questions, they can resort to Contact us module.

### Search

In order to help users efficiently find out the disease relationships surrounding one of the 108 diseases, we provide five searching strategies, via disease name, disease gene, disease drug, tissue name and GSE ID, respectively. In addition, users can search for several diseases or GSE IDs or tissues by using unlimited batch, that is, selecting multiple keywords.

**Fig. 2:**
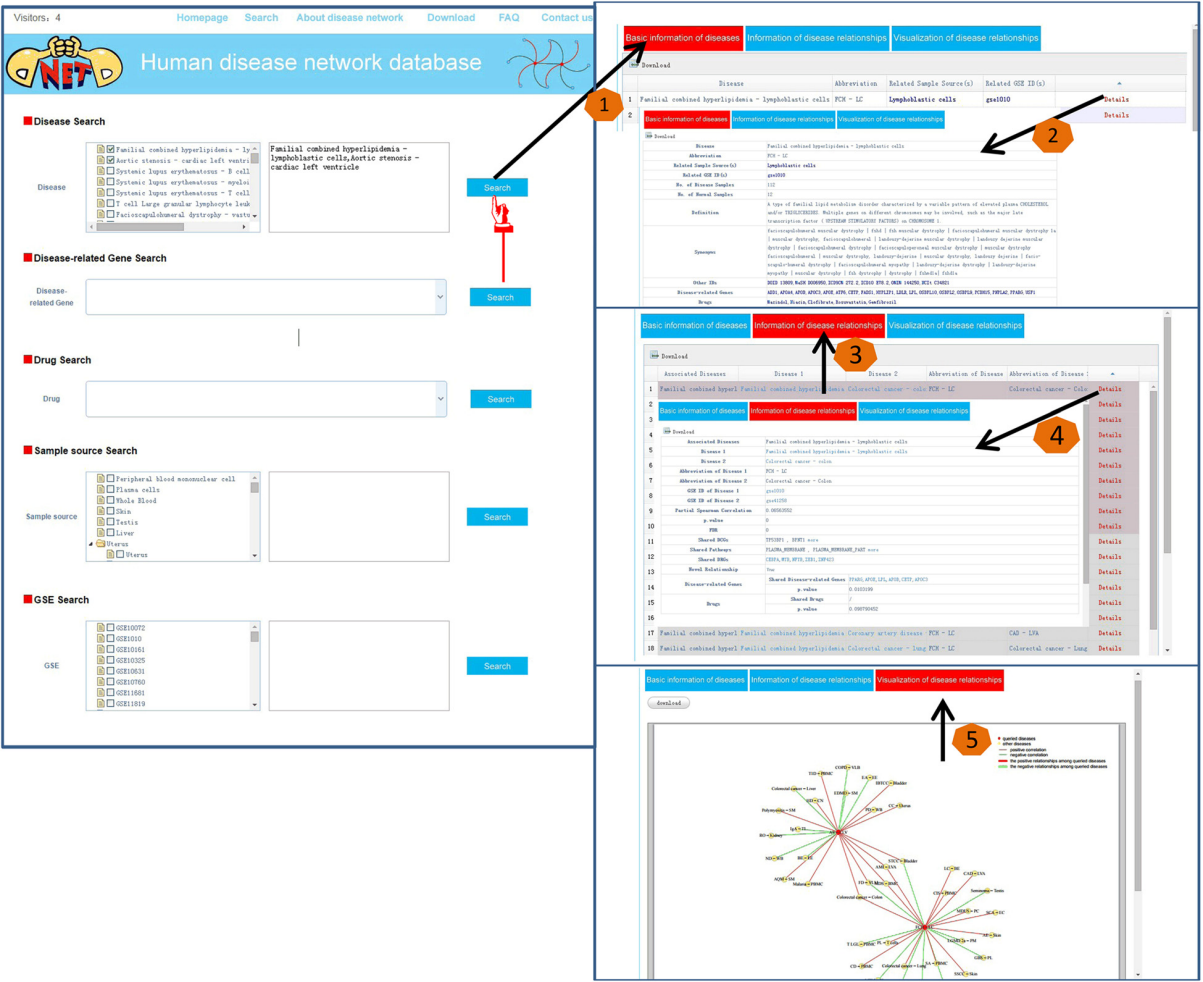
An example of Search module

Among the five searching strategies, searching by disease name is the core function. The query results contain three parts including basic information of disease, information of disease relationships and the diagram of disease relationships (Fig. 2). The basic information of disease includes abbreviate name, definition, related GSE, sample source, number of samples, other IDs of disease (including disease IDs in DO, MeSH, ICD10, ICD9, OMIM and NCIt) which can be connected to corresponding databases, disease-related genes and drugs. If only one disease is queried, DNetDB displays the relationships between the queried disease and its similar diseases. If two or more diseases are queried, the relationships within queried diseases, as well as those between the queried diseases and the other diseases are retrieved, which are displayed with different colors. For each disease relationship, the DNetDB offers the eleven items described above. Genes, pathways and drugs in the result table can be linked to Genecards, MSigDB, Drugbank, respectively. Through above exhibitions, users can capture the shared DCGs and the shared pathways of any disease pair, which help to explore the common dysfunctional regulation mechanisms. Finally, the disease relationships are shown in graph with nodes and lines for facilitating observation. Red nodes represent queried diseases; yellow nodes represent similar diseases of queried diseases. Red lines represent positively correlated disease relationships; green lines represent negatively correlated relationships. Broad lines represent disease relationships among queried diseases; fine lines represent disease relationships between queried diseases and other diseases (Fig. 2).

Besides, searching via disease gene and disease drug, are also implemented. The disease(s) associated with queried gene or drug is marked firstly; then DNetDB seeks and exports disease relationships around the marked disease(s). This helps user perform drug repositioning and explore pathogenesis since pathogenesis and drug clinical application can be appropriated from one disease to its similar ones.

In our companion work [30], we found both the type of disease and the type of affected tissue influence disease similarity. We therefore supply a fourth searching category, by sample tissue, to check the relationships between the diseases which originate from a certain tissue. The searching results are also showed in the same manner as the results of searching by disease names.

When searching via GSE IDs, the GSE IDs would be converted into disease names. Besides the common results like directly searching via disease names, the basic information of GSEs, including the titles, PMID of PubMed, are also included.

### Download

The Download module provides five comprehensive files to be downloaded, including basic information of the 86 GSEs, basic information of the 108 diseases, the 1,326 disease pairs, and the dC value tables as well. And a specific searching result can also be downloaded.

## Discussion

Disease similarity are of great interest because this knowledge enhances our understanding of disease etiology and pathogenesis [1]. The currently available disease similarity databases [22–24] collect disease relationships which were estimated according to clinical information, phenotypic characteristics, and known disease-related genes, therefore have limited chance to contain novel disease relationships or support discovering novel knowledge. In transcriptomic domain, differential coexpression analysis has been proved to be able to unravel dysfunctional regulation mechanism. By using a differential coexpression analysis method previously developed in our DCGL v2 R package [26–29], we identified 1,326 disease relationships among 108 diseases based on gene expression data [30]. The 1,326 disease relationships and their relevant information were collected in the human disease network database (DNetDB), which can be visited at http://app.scbit.org/DNetDB/#.

The database provides abundant information including not only disease definition, six types of IDs for disease (including disease IDs in DO, MeSH, ICD10, ICD9, OMIM and NCIt), GSE IDs, samples size (the number of disease samples the health samples), but also common DCGs, pathways, DRGs, disease-related genes and drugs for each disease pair, as well as graphical display of disease relationships. The common DCGs and DRGs which reflect the common dysfunctional regulation mechanism are extracted and presented in our database. In addition, the details of common DCGs and DRGs in the two diseases of a disease pair could also be displayed, such as the proportion of common DCGs, dC values of common DCGs, and number of common DRGs.

By providing searching strategies via disease gene and disease drug, DNetDB greatly supports drug repositioning and pathogenesis exploration, since user can appropriate the pathogenesis and drug application from one disease to its similar diseases. In our companion paper [30], we have proved that if a drug is able to successfully treat disease A in our disease network, it tends to treat A-linked diseases too. For example, in DNetDB, Psoriasis and T-cell polymphoytic leukaemia is a disease pair with a p-value of 0.035, by the hypergeometric test for shared drugs. Drugs for Psoriasis include Prednisone, Methotrexate, Tazarotene and etc., a total of 19. Drugs for T-cell polymphoytic leukaemia are Prednisone, Methotrexate, Rituximab and etc., a total of 14. Their common drugs are Prednisone and Methotrexate. Methotrexate, an antimetabolite and antifolate drug, is recorded in American Hospital Formulary Service (ASHP) drug information 2004 for treatment of both polymphoytic leukaemia and Psoriasis [42]. Accordingly, we propose that the other drugs for Psoriasis maybe also have the potential to treat polymphoytic leukaemia and vice versa. Another interesting example is Parkinson’s disease and Influenza A. Amantadine hydrochloride (trade name Symmetrel, by Endo Pharmaceuticals) has been approved for treatment of both Influenza A and Parkinson’s disease [43]. Similarly, we propose that other drugs for Parkinson, for example, Droxidopa and Ioflupane I, and the other drugs for Influenza A, for example, Zanamivir, Rimantadine and Thymalfasin, are also worth for further exploration for their potential in drug repositioning.

In conclusion, DNetDB is the first database which presents disease relationships from the viewpoint of gene regulation mechanism. DNetDB records common dysfunctional regulation mechanism, common disease-related genes and drugs of disease pairs. In this way, DNetDB has the capability to support pathogenesis exploration, drug repositioning and drug development.

## Conclusion

DNetDB is the first disease similarity database from the viewpoint of dysfunctional regulation mechanism. It provides an easy-to-use web interface to search and browse disease relationships through disease name(s), disease gene, drug, GSE ID(s), and tissue (s). DNetDB facilitates the study of disease relationships and provides insightful clues to investigate diseases’ etiology and pathogenesis, perform drug repositioning, and design novel therapeutic interventions.

### Availability and requirements

DNetDB is freely available for use at http://app.scbit.org/DNetDB/#.

Operating Systems: DNetDB is accessed from a browser; therefore, it is platform-independent.

Browsers: DNetDB is extensively tested with browsers Internet Explorer, Google Chrome and Mozilla Firefox.

## Additional file

Additional file 1: Additional analysis and concepts explanation. This file contains 1) comparison of DNetDB and the results of differential expression analysis (DEA-) based method ; 2) comparison of DNetDB and traditional disease classification; 3) negative disease relationships and 4) DCp and DCe. (DOCX 6926 kb)

## Abbreviations

dC: differential coexpression value
DCG: differentially coexpressed gene
DCL: differentially coexpressed link
DRG: differentially regulated gene
DRG: differentially regulated gene
DRL: differentially regulated link
DRL: differentially regulated links
