# Supplementary material for: DNetDB: The human disease network database based on dysfunctional regulation mechanism
Source: BMC Syst Biol. 2016 May 21;10:36. doi: 10.1186/s12918-016-0280-5 (PMC4875653; doi:10.1186/s12918-016-0280-5)
Supplement: Additional file 1: — Additional analysis and concepts explanation. This file contains 1) comparison of DNetDB and the results of differential expression analysis (DEA-) based method ; 2) comparison of DNetDB and traditional disease classification; 3) negative disease relationships and 4) DCp and DCe. (DOCX 6926 kb) [file 12918_2016_280_MOESM1_ESM.docx]

DNetDB: The human disease network database based on dysfunctional regulation mechanism

Jing Yang, Su-Juan Wu, Shao-You Yang, Jia-Wei Peng, Shi-Nuo wang, Fu-Yan Wang, Yu-Xing Song, Ting Qi, Yi-Xue Li and Yuan-Yuan Li

1. **Comparison of DNetDB and the results of differential expression analysis (DEA-) based method**

Both our proposed method and the compared method were designed to explore disease similarities based on gene expression data. The difference is, our method applied a new emerging strategy, differential coexpression analysis (DCEA), while the compared method adopted the traditional differential expression analysis (DEA).

In order to compare the DNetDB and results of DEA-based method, we selected 954 GSE datasets (GSE short for GEO series) designed for human studies using Affymetrix U133A chip (i.e. GPL96), the most commonly used platform, from GEO (http://www.ncbi.nlm.nih.gov/geo/). We then picked out 106 GSEs which 1) were assigned to human disease condition and corresponding normal condition, 2) had more than five samples in each condition, and 3) came from fresh organs (excluding cell lines). We downloaded raw data (CEL files) of each sample, controlled and removed low quality samples using affy ([Gautier, et al., 2004](#_ENREF_2)) and affyQCReport ([Parman and Halling, 2013](#_ENREF_11)) packages, and finally retained 86 GSE datasets involving 4,403 samples for 108 diseases.

We identified 1,326 significant disease-disease links (DDLs) among the 108 diseases by using DCEA-based method. Simultaneously, we replaced the dC value in our similarity measurement with differential expression level, the log of Fold Change, and obtained 1,583 DEA-based DDLs.

We tested whether the 1,326 disease pairs can significantly share disease-related genes or drugs with Hypergeometric test since similar diseases tend to share similar pathogenesis, and thus have the potential to be treated by common drugs ([Suthram, et al., 2010](#_ENREF_13)), We found 81% and 47% of 1,326 DDLs significantly share common disease-related genes and drugs. And the proportion, 81% and 47%, are also significantly higher than in random (p-values were 0.009 for disease-related genes and 0.023 for disease drugs with one-sided Fisher exact test). However, one-sided Fisher’s exact tests showed that the DEA-based DDLs did not significantly share disease-related genes (p-value 0.229) and disease drugs (p-value 0.596).

According to the percentage of the disease pairs which significantly share drugs (Figure S1, color depth of each region), it was found that DCEA-based DDLs (DCEA_sig, including “773” region, “162” region and “391” region in Figure S2) share drugs much more remarkably than DEA-based DDLs (DEA_sig in Figure S1), DEA-based non-significant pairs (DEA_nonsig in Figure S1), and DCEA-based non-significant pairs (DCEA_nonsig in Figure S1) in order. At the same time, non-significant disease pairs identified by DCEA-based method (DCEA_nonsig, including “2,554” region, “511” region and “1,387” region in Figure S1) have the lowest percentages of the disease pairs which significantly share drugs. However, among the DEA-based DDLs (DEA_sig, including “910” region, “162” region and “551” region in Figure S2), the 551 DDLs which are non-significant disease pairs according to DCEA-based method have the lowest percentage of the DDLs which significantly share drugs; while, out of the DEA-based non-significant pairs (DEA_nonsig, including “2,417” region, “391” region, “1,387” region in Figure S1), the 391 non-significant pairs which are DDLs according to DCEA-based method have the highest percentage of disease pairs sharing drugs. In this way, the disease network based on DCEA proved to be more relevant to pathogenesis than that based on DEA. Figure S1 clearly captures the potential false positive and false negative disease pairs identified by DEA-based strategy, and explains why the DCEA-based strategy outperformed DEA-based strategy.





**Figure S1. Comparison of two types of disease networks which were identified based on DCEA-based method and DEA-based method.** DCEA_sig and DCEA_nonsig denote significant and non-significant disease pairs which were identified by DCEA-based method. DEA_sig and DEA_nonsig denote significant and non-significant disease pairs which were identified by DEA-based method. Meanwhile, the depth of color in every region represents the percentages of disease pairs which significantly share disease drugs.

In order to compare the relevance of DCEA-based method and DEA-based method, we specially extracted 32 cancer datasets from our 108 datasets. Since cancer progression requires the coordination of cancer genes which involve common tumor activators (such as Ras and Myc) and tumor suppressors (such as p53 and PTEN), we simultaneously calculated dC values and the log values of Fold Change of Ras (NRAS, KRAS, HRAS and MRAS), Myc, p53 and PTEN in each cancer type, and proposed that the more relevant the measurement, the more coherent the value across the various cancer genes. It was found that gene differential coexpression values (dCs) in the 32 cancer types are coincident across the seven cancer genes; in contrast, the log of Fold Change didn’t display any significant pattern. This result further supports the rationality of our DCEA-based analysis strategy.

1. **Comparison of DNetDB and traditional disease classification**

In order to study the consistency of DNetDB with previous knowledge on disease classification, we carried out the following analyses. We marked the 108 diseases in our disease network with their category names in traditional disease classification depository, such as MeSH, ICD-10 and DO, and thus the disease network were divided into several sub-networks according to category markers. In order to check if the diseases from the same category are inclined to form compact sub-network in our disease network, we applied a metric, within-network distance (WD), to estimate the relational closeness of each sub-network ([Li and Agarwal, 2009](#_ENREF_5)). According to Li et al.’s work, the mean shortest path length among all links in a network was defined as within-network distance (WD) in order to describe the relational closeness of a network (Eq. S1) ([Li and Agarwal, 2009](#_ENREF_5)).

$WD_{c}=\frac{\sum d(i,j)}{k}, i,j\in c$ (Eq. S1)

Where k denotes the total number of links in the network, and d (i, j) denotes the shortest path between vertex i and j. The smaller the WD value, the greater the network compactness. Theoretically when WD=1, the network is fully connected, displaying as a complete graph.

When the WD value of a sub-network is smaller than that of the whole network, the diseases in the sub-network, or within the category, are proposed to lie closer to each other. We found that most of the within-category diseases form more compact sub-networks than the background. Our disease network was proved to be basically compatible with traditional disease classification systems. We turned to check the 1,326 significant disease relationships (DDLs) in our disease network individually to see if they are consistent with the previous knowledge in MeSH, ICD-10 and DO. It was found that for 566 DDLs (~43%), the disease pair share at least one common disease category. While, the left 760 DDLs (~57%) are supposed to be novel disease relationships, among which 82.13% significantly share disease-related genes or drugs.

1. **Negative disease relationships**

When disease A and A’ form a negative link, the patient with disease A tends to be protected from having disease A’ and vice versa, which is probably due to the inversely regulated biological processes involved in the negatively correlated diseases ([Hu and Agarwal, 2009](#_ENREF_3)). For example, in 1,326 DDLs, Muscular dystrophy is negatively correlated to some cancers probably because Muscular dystrophy involves the inhibition of cell overgrowth while cancer involves the activation of cell overgrowth. In agreement with Liu et al. opinion, the disease similarity study based on omics data has more chance to find negatively correlated diseases than based on clinical symptom information or gene-phenotype data, because text-mining techniques for clinical symptom information cannot process negative language and gene-phenotype data include disease causal information rather than preventive information ([Liu, et al., 2009](#_ENREF_7)). The proportion of negative links in our data (~40%) is even much higher than Hu et. al’s report (~30%) which adopted differential expression based method to calculate disease similarity([Hu and Agarwal, 2009](#_ENREF_3)). We found that 25% of Hu et. al’s negative links were also sorted out in our data. Since DCEA has more potential to discover regulation mechanisms than DEA does, we propose that the negative links which are not included in Hu et. al’s work also deserve further investigation. By tracing the differential coexpression properties of a negatively correlated disease pair, one may obtain useful hints for explaining the underlying mechanisms of the mutual exclusion of the two diseases.

Furthermore, since negatively correlated diseases, say disease A and A’, involve inversely regulated biological processes, we also proposed that an anti-A drug may have an undesired property of inducing disease A’ when the drug is inversing its target processes. Taking Crohn’s disease and its therapeutic drug, infliximab, as an example, Crohn’s disease is negatively connected with T-cell source of chronic lymphocytic leukemia (correlation coefficient -0.15, at top 5%) and Melanoma (correlation coefficient -0.05, at top 50%) in our data; Infliximab, a [chimeric](http://en.wikipedia.org/wiki/Chimeric_protein) [monoclonal antibody](http://en.wikipedia.org/wiki/Monoclonal_antibody) against tumor necrosis factor alpha ([TNF](http://en.wikipedia.org/wiki/TNF_alpha)-α), is usually used for treatment of inﬂammatory bowel disease (IBD) such as Crohn’s disease ([FDA, 1998-8-24](#_ENREF_1)). In 2006, the Food and Drug Administration (FDA) issued a warning for inﬂiximab given its potential association with the development of Hepatosplenic T-cell lymphoma which is a subtype of T-cell source of chronic lymphocytic leukemia ([Mackey, et al., 2007](#_ENREF_10)). This phenomenon was also observed in other independent studies ([Kotlyar, et al., 2011](#_ENREF_4); [Mackey, et al., 2009](#_ENREF_9); [Shale, et al., 2008](#_ENREF_12)). Similarly, a case-control study showed an increased risk of melanoma with anti-TNF treatment in IBD patients ([Long, et al., 2012](#_ENREF_8)). We believe that the differential coexpression properties of these negatively correlated diseases could help to explore the underlying mechanisms and improve the therapeutic applications.

1. **DCp and DCe**

DCp ([Liu, et al., 2010](#_ENREF_6); [Yang, et al., 2013](#_ENREF_14)), Differential Coexpression Profile, was developed to identify differentially coexpressed genes (DCGs) via estimating the degree of correlation change between disease pairs (Eq. S2).

$dC_{i}(DCp)=\sqrt{\frac{(x_{i1}-y_{i1})^{2}+(x_{i2}-y_{i2})^{2}+...+(x_{in}-y_{in})^{2}}{n}}$ (Eq. S2)

Where $X=\{x_{i1},x_{i2},...,x_{in}\}$ and $Y=\{y_{i1},y_{i2},...,y_{in}\}$ indicate coexpression value of gene i with their n associated genes for two conditions (for example, disease samples and normal samples). This measure captures the average coexpression change between a gene and its neighbors. The dC value can be used to rank genes. To evaluate the statistical significance of dC, we perform a permutation test, in which we randomly permute the disease and normal conditions of the samples, calculate new coexpression value of gene i with their n neighbors, filter gene pairs based on the new coexpression value, and calculate the dC statistics. The sample permutation is repeated 1000 times, and a large number of permutation dC statistics form an empirical null distribution. The p-value for each gene can then be estimated. Accordingly, DCGs can be obtained when user given a cutoff of p-value.

DCe ([Liu, et al., 2010](#_ENREF_6); [Yang, et al., 2013](#_ENREF_14)), Differential Coexpression Enrichment, was developed to identify both DCGs and differentially coexpressed links (DCLs). Here we mainly introduce the function of DCe for identifying DCLs. As shown in Figure S3, we used Limit Fold Change model applied to identify DCLs.


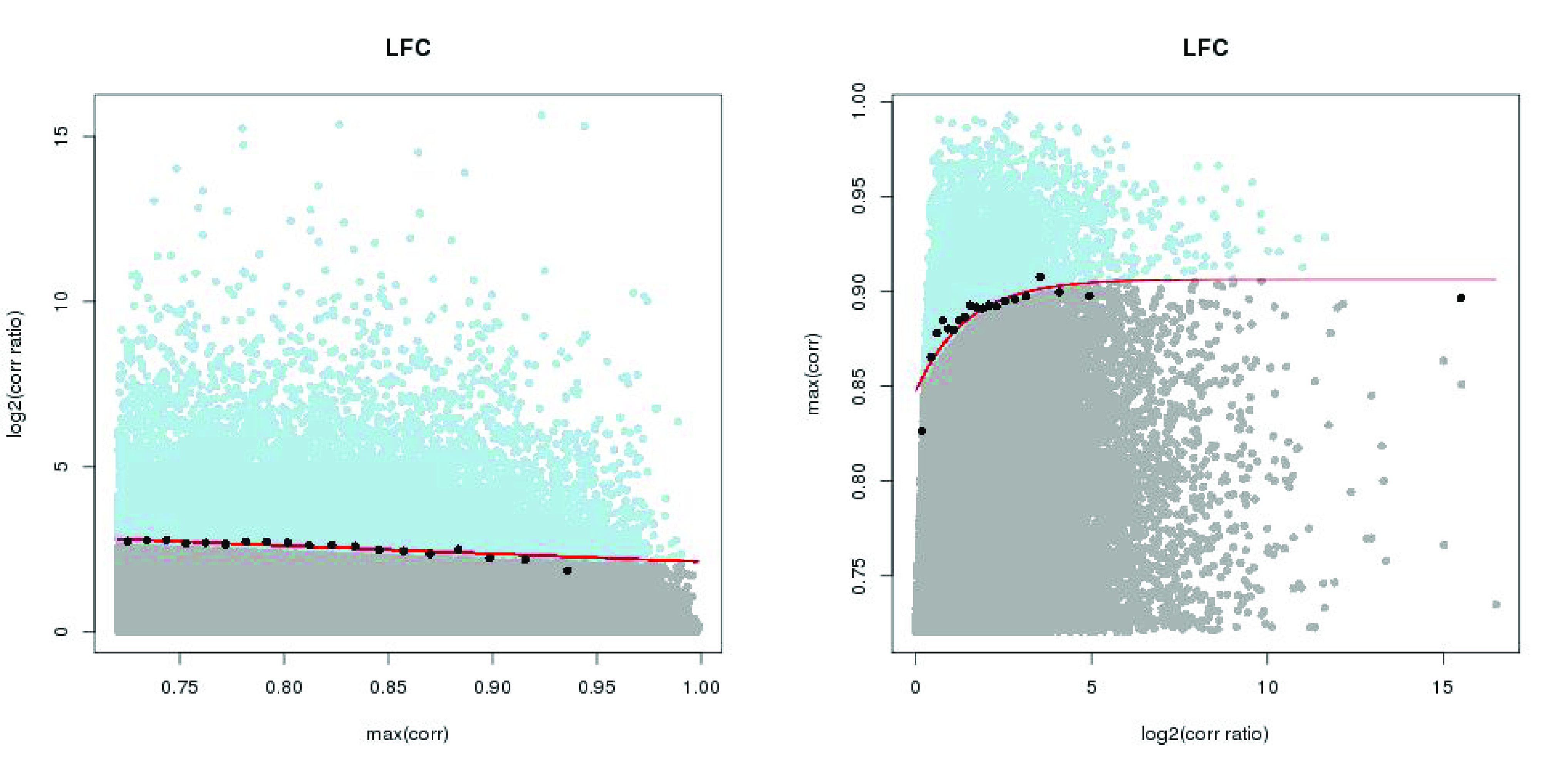


**Figure S3. Limit Fold Change model applied to identify DCLs.** Left, same signed links; right, differently signed links.

Each point represents a gene pair or a link characterized by its log correlation ratio and maximum absolute correlation value. A curve (red) y = a + (b/x) is used to fit the boundary outliers (black dots) determined by fraction δ, and points (blue) lying above the fitted curves are considered DCLs.

**References**

FDA (1998-8-24) Infliximab Product Approval Information - Licensing Action, *Drugs@FDA US Food and Drug Administration (FDA)*.

Gautier, L.*, et al.* (2004) affy—analysis of Affymetrix GeneChip data at the probe level, *Bioinformatics*, **20**, 307-315.

Hu, G. and Agarwal, P. (2009) Human disease-drug network based on genomic expression profiles, *PLoS One*, **4**, e6536.

Kotlyar, D.S.*, et al.* (2011) A systematic review of factors that contribute to hepatosplenic T-cell lymphoma in patients with inflammatory bowel disease, *Clin Gastroenterol Hepatol*, **9**, 36-41 e31.

Li, Y. and Agarwal, P. (2009) A pathway-based view of human diseases and disease relationships, *PLoS One*, **4**, e4346.

Liu, B.H.*, et al.* (2010) DCGL: an R package for identifying differentially coexpressed genes and links from gene expression microarray data, *Bioinformatics*, **26**, 2637-2638.

Liu, Y.I., Wise, P.H. and Butte, A.J. (2009) The "etiome": identification and clustering of human disease etiological factors, *BMC Bioinformatics*, **10 Suppl 2**, S14.

Long, M.D.*, et al.* (2012) Risk of melanoma and nonmelanoma skin cancer among patients with inflammatory bowel disease, *Gastroenterology*, **143**, 390-399 e391.

Mackey, A.C.*, et al.* (2009) Hepatosplenic T cell lymphoma associated with infliximab use in young patients treated for inflammatory bowel disease: update, *J Pediatr Gastroenterol Nutr*, **48**, 386-388.

Mackey, A.C.*, et al.* (2007) Hepatosplenic T cell lymphoma associated with infliximab use in young patients treated for inflammatory bowel disease, *J Pediatr Gastroenterol Nutr*, **44**, 265-267.

Parman, C. and Halling, C. (2013) affyQCReport: A Package to Generate QC Reports for Affymetrix Array Data.

Shale, M.*, et al.* (2008) Hepatosplenic T cell lymphoma in inflammatory bowel disease, *Gut*, **57**, 1639-1641.

Suthram, S.*, et al.* (2010) Network-based elucidation of human disease similarities reveals common functional modules enriched for pluripotent drug targets, *PLoS Comput Biol*, **6**, e1000662.

Yang, J.*, et al.* (2013) DCGL v2.0: An R Package for Unveiling Differential Regulation from Differential Co-expression, *PLoS One*, **8**, e79729.

Yu, H.*, et al.* (2011) Link-based quantitative methods to identify differentially coexpressed genes and gene pairs, *BMC Bioinformatics*, **12**, 315.
